# Treatment of large intracranial aneurysms using the Woven EndoBridge (WEB): a propensity score-matched analysis

**DOI:** 10.1007/s10143-024-02527-5

**Published:** 2024-07-31

**Authors:** Basel Musmar, Hamza Adel Salim, Nimer Adeeb, Assala Aslan, Bahaa Aljeradat, Jose Danilo Bengzon Diestro, Rachel M. McLellan, Oktay Algin, Sherief Ghozy, Mahmoud Dibas, Sovann V. Lay, Adrien Guenego, Leonardo Renieri, Nicole M. Cancelliere, Joseph Carnevale, Guillaume Saliou, Panagiotis Mastorakos, Kareem El Naamani, Eimad Shotar, Kevin Premat, Markus Möhlenbruch, Michael Kral, Justin E. Vranic, Charlotte Chung, Mohamed M. Salem, Ivan Lylyk, Paul M. Foreman, Jay A. Vachhani, Hamza Shaikh, Vedran Župančić, Muhammad U. Hafeez, Joshua Catapano, Muhammad Waqas, Vincent M. Tutino, Mohamed K. Ibrahim, Marwa A. Mohammed, M. Ozgur Ozates, Giyas Ayberk, James D. Rabinov, Yifan Ren, Clemens M. Schirmer, Mariangela Piano, Anna L. Kühn, Caterina Michelozzi, Stéphanie Elens, Robert M. Starke, Ameer Hassan, Mark Ogilvie, Anh Nguyen, Jesse Jones, Waleed Brinjikji, Marie T. Nawka, Marios Psychogios, Christian Ulfert, Julian Spears, Brian T. Jankowitz, Jan-Karl Burkhardt, Ricardo A. Domingo, Thien Huynh, Juan Carlos Martinez-Gutierrez, Muhammed Amir Essibayi, Sunil A. Sheth, Gary Spiegel, Rabih Tawk, Boris Lubicz, Pietro Panni, Ajit S. Puri, Guglielmo Pero, Erez Nossek, Eytan Raz, Monika Killer-Oberfalzer, Christoph J. Griessenauer, Hamed Asadi, Adnan Siddiqui, Allan Brook, David Altschul, Andrew F. Ducruet, Felipe C. Albuquerque, Robert W. Regenhardt, Christopher J. Stapleton, Peter Kan, Vladimir Kalousek, Pedro Lylyk, Srikanth Boddu, Jared Knopman, Mohammad A. Aziz-Sultan, Stavropoula I. Tjoumakaris, Frédéric Clarençon, Nicola Limbucci, Hugo H. Cuellar-Saenz, Pascal M. Jabbour, Vitor Mendes Pereira, Aman B. Patel, Adam A. Dmytriw

**Affiliations:** 1 Department of Neurosurgery and Interventional Neuroradiology, Louisiana State University, Shreveport, LA USA; 2 Division of Diagnostic and Therapeutic Neuroradiology, Department of Radiology, St. Michael’s Hospital, University of Toronto, Toronto, ON Canada; 3 Neuroendovascular Program, Massachusetts General Hospital & Brigham and Women’s Hospital, Harvard University, Boston, MA USA; 4 Department of Radiology, Medical Faculty of Ankara University, Ankara, Turkey; 5 Department of Radiology and Neurosurgery, Mayo Clinic, Rochester, MN USA; 6 Service de Neuroradiologie Diagnostique Et Thérapeutique, Centre Hospitalier de Toulouse, Hôpital Purpan, Toulouse, France; 7 Service de Neuroradiologie Interventionnelle, Hôpital Universitaire Erasme, Bruxelles, Belgique; 8 Interventistica Neurovascolare, Ospedale Careggi Di Firenze, Florence, Italy; 9 Neurosurgery & Interventional Neuroradiology, New York Presbyterian Hospital, Weill Cornell School of Medicine, New York, NY USA; 10 Service de Radiodiagnostic Et Radiologie Interventionnelle, Centre Hospitalier Vaudois de Lausanne, Lausanne, Switzerland; 11 Department of Neurosurgery, Thomas Jefferson University, Philadelphia, PA USA; 12 Department de Neuroradiologie, Hôpital Pitié-Salpêtrière. Université Sorbonne, Paris, France; 13 Sektion Vaskuläre Und Interventionelle Neuroradiologie, Universitätsklinikum Heidelberg, Heidelberg, Germany; 14 Department of Neurosurgery, Christian Doppler University Hospital & Institute of Neurointervention, Paracelsus Medical University, Salzburg, Austria; 15 Department of Radiology & Neurosurgery, NYU Langone Health Center, New York, NY USA; 16 Department of Neurosurgery, University of Pennsylvania Medical Center, Philadelphia, PA USA; 17 Equipo de Neurocirugía Endovascular y Radiología IntervencionistaClínica La Sagrada Familia, Buenos Aires, Argentina; 18 Neurosurgery Department, Orlando Health Neuroscience and Rehabilitation Institute, Orlando, FL USA; 19 Department of Radiology & Neurosurgery, Cooper University Health Care, Cooper Medical School of Rowan University, Camden, NJ USA; 20 Subdivision of Interventional Neuroradiology, Department of Radiology, Clinical Hospital Center ‘Sisters of Mercy’, Zagreb, Croatia; 21 Department of Neurosurgery, UTMB and Baylor School of Medicine, Houston, TX USA; 22 Department of Neurosurgery, Barrow Neurological Institute, Phoenix, AZ USA; 23 Department of Neurosurgery, State University of New York at Buffalo, Buffalo, NY USA; 24 Interventional Radiology and Neurointerventional Services, Department of Radiology, Austin Health, Melbourne, VIC Australia; 25 Department of Neurosurgery and Radiology, Geisinger Hospital, Danville, PA USA; 26 Interventistica Neurovascolare, Ospedale Niguarda Cà Granda, Milano, Italy; 27 Department of Neurointerventional Radiology, UMass Memorial Hospital, Worcester, MA USA; 28 Interventistica Neurovascolare, Ospedale San Raffaele, Milano, Italy; 29 Deparment of Neurosurgery, University of Miami, Miami, FL USA; 30 Deparment of Neuroscience, Valley Baptist Neuroscience Institute, Harlingen, TX USA; 31 Deparment of Neurosurgery and Radiology, University of Alabama at Birmingham, Birmingham, AL USA; 32 Department of Interventional Neuroradiology, Interventional Neuroradiology, University Hospital of Basel, Basel, Switzerland; 33 Department of Diagnostic and Interventional Neuroradiology, University Medical Center Hamburg-Eppendorf, Hamburg, Germany; 34 Department of Radiology and Neurosurgery, Mayo Clinic, Jacksonville, FL USA; 35 Department of Radiology, Neurology, and Neurosurgery, University of Texas Health Science Center at Houston, Houston, TX USA; 36 Department of Neurological Surgery and Montefiore-Einstein Cerebrovascular Research Lab, Montefiore Medical Center, Albert Einstein College of Medicine, Bronx, NY USA; 37 Neurosurgery Department, Ankara Yildirim Beyazit University, Ankara, Turkey

**Keywords:** Aneurysms, Intracranial, WEB, Woven EndoBridge, Treatment

## Abstract

The Woven EndoBridge (WEB) device is primarily used for treating wide-neck intracranial bifurcation aneurysms under 10 mm. Limited data exists on its efficacy for large aneurysms. We aim to assess angiographic and clinical outcomes of the WEB device in treating large versus small aneurysms*.* We conducted a retrospective review of the WorldWide WEB Consortium database, from 2011 to 2022, across 30 academic institutions globally. Propensity score matching (PSM) was employed to compare small and large aneurysms on baseline characteristics. A total of 898 patients were included. There was no significant difference observed in clinical presentations, smoking status, pretreatment mRS, presence of multiple aneurysms, bifurcation location, or prior treatment between the two groups. After PSM, 302 matched pairs showed significantly lower last follow-up adequate occlusion rates (81% vs 90%, *p* = 0.006) and higher retreatment rates (12% vs 3.6%, *p* < 0.001) in the large aneurysm group. These findings may inform treatment decisions and patient counseling. Future studies are needed to further explore this area.

## Introduction

The natural course of large intracranial aneurysms (≥ 10 mm) is typically unfavorable, and early intervention is generally recommended [1]. According to a study from Japan, the yearly risk of rupture is 4.37% for aneurysms between 10 and 24 mm in size and 33.4% for those larger than 24 mm [2]. As surgical treatment of those aneurysms could be challenging and may cause significant complications [3, 4], several endovascular techniques had evolved and are gradually becoming more popular as a minimally invasive alternative to open surgery [5, 6].

The Woven EndoBridge (WEB) is a self-expanding device made of nitinol that disrupts blood flow within the aneurysm, acting as an intrasaccular flow diverter [7]. It is a viable option for treating complex aneurysms that cannot be treated with standard embolization devices or techniques, particularly wide-neck bifurcation aneurysms [8, 9]. The device is available in different models and sizes and has undergone changes in the last years [10]. While the WEB has shown good short-and mid-term results, long-term follow-up data are limited, especially for larger and complex aneurysms [10].

In this study, we conducted a multicenter cohort study to compare the treatment outcomes and complications between small (maximum diameter < 7.5 mm) and large (≥7.5 mm) intracranial aneurysms using the WEB device.

## Methods

### Patient sample

The WorldWide WEB Consortium is a synthesis of retrospective databases at 30 academic institutions in North America, South America, and Europe. A standardized data sheet was used to identify patients with intracranial aneurysms treated with WEB device, spanning from January 2011 and December 2022. All consecutive adult patients (age ≥18 years) with both ruptured and unruptured saccular aneurysms in all locations that were treated with the WEB were included. Other aneurysms shapes, including fusiform and blister aneurysms, are not suitable for WEB device placement and, thus, were not included. The selection of aneurysms with suitable size and neck angle for WEB device placement was at the discretion of each interventionalist and was not enforced by the study protocol. The following information was collected using the standardized datasheet: patient demographics (age, gender, smoking status, presentation, modified Rankin Score (mRS), and use of antiplatelets), aneurysm characteristics (side, size, width, location, multiple aneurysms, daughter sac, branch arising from aneurysm, and prior treatment), procedural details (procedure date, type of access, length of procedure, fluoroscopy time, WEB size, and posttreatment antiplatelets), complications (type, timing, location, symptoms, duration, and additional treatment), angiographic outcomes (length of imaging follow-up, immediate flow stagnation, immediate and follow-up occlusion rate, device compaction, fate of branches arising from aneurysm, and retreatment), and functional outcomes (length of clinical follow-up, mRS at last follow-up, and mortality). Patients who presented with ruptured aneurysms were excluded. Also, patients who needed adjunctive devices post-WEB were excluded. Aneurysms were then categorized based on its maximum diameter into two groups: 1- large aneurysms (≥7.5 mm); and 2- small aneurysms (< 7.5 mm). Institutional review board approval was obtained at all centers. No identifiable patient information was presented in the study and, thus, informed consent was not required.

### Angiographic outcomes

The angiographic outcome was assessed using digital subtraction angiography (DSA), MR angiography, or CT angiography. Aneurysm occlusion after treatment, both immediately and at last follow-up, was categorized using the three-point occlusion scale: complete occlusion (Raymond Roy (RR) 1), neck remnant (RR2, and aneurysm remnant (RR3) [11]. Adequate occlusion was defined as either complete occlusion or neck remnant with lack of an aneurysm remnant.

### Functional outcomes and complications

Functional outcome was assessed using mRS at the last follow-up. Independent functional status was defined with a mRS score of 0–2.

Thromboembolic complications occurring from the date of the procedure up to the last follow-up were recorded. Intra-procedural thromboembolic complications were identified on DSA as either thrombus formation, slow filling of a previously normal filling vessel, or complete vessel occlusion. Post-procedural thromboembolic complications were identified using a combination of clinical and radiographic findings. Post-procedural imaging was performed at the discretion of the individual institutions. Routine screening for clinically silent infarcts was not consistently performed. An ischemic complication was considered symptomatic if there were patient-reported symptoms or clinical signs attributable to thromboembolism; this included transient or resolving signs and symptoms. Hemorrhagic complications were identified intra-operatively as contrast extravasation on DSA or post-procedure imaging. Hemorrhages were counted as symptomatic if the patient-reported symptoms or demonstrated signs attributable to hemorrhage. Other complications included intraprocedural device deployment issues, air embolism, and vascular access complications. Complications were considered permanent if still present at a 3-month follow-up.

### Statistical analysis

The statistical analysis was conducted using R studio version 4.2.2. Categorical variables were summarized as frequencies with percentages and compared using the × 2 test or ordinal mixed effect logistic regression for ordinal variables. Continuous variables were summarized as medians with interquartile ranges (IQR) and compared using the Mann–Whitney U test. Variables that were recognized as possible confounders were selected to be included in propensity score matching (PSM). To achieve balance Hunt Hess score variable was added to the matching process, and the fixed ratio 1:1 PSM optimal matching method was used. Results were considered statistically significant if they had a P value of 0.05 or less.

**Fig. 1:**
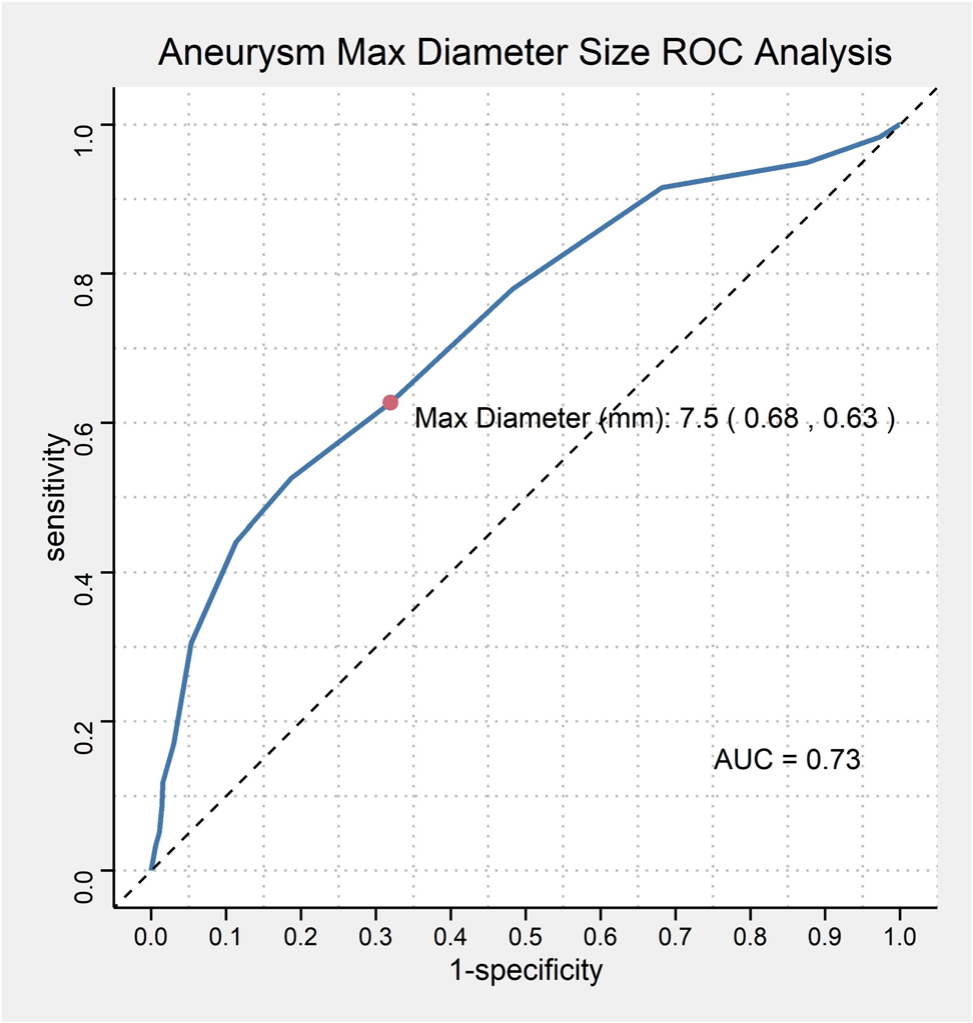
Flowchart shows the inclusion for patients and aneurysms in this study

ROC curves were generated to determine the optimal cut-off points for aneurysm size based on retreatment rates. The closest topleft criterion was employed to calculate the optimal cut-off points, which was found to be 7.5. The area under the curve (AUC) was 0.73, with a sensitivity of 0.68 and specificity of 0.63 (Fig. 1).

Multiple imputations were conducted with 50 imputations, followed by mixed-effect logistic regression and ordinal logistic regression. Stepwise selection was used to select the variables to be included for each outcome model.

## Results

### Patient and aneurysm characteristics

**Fig. 2:**
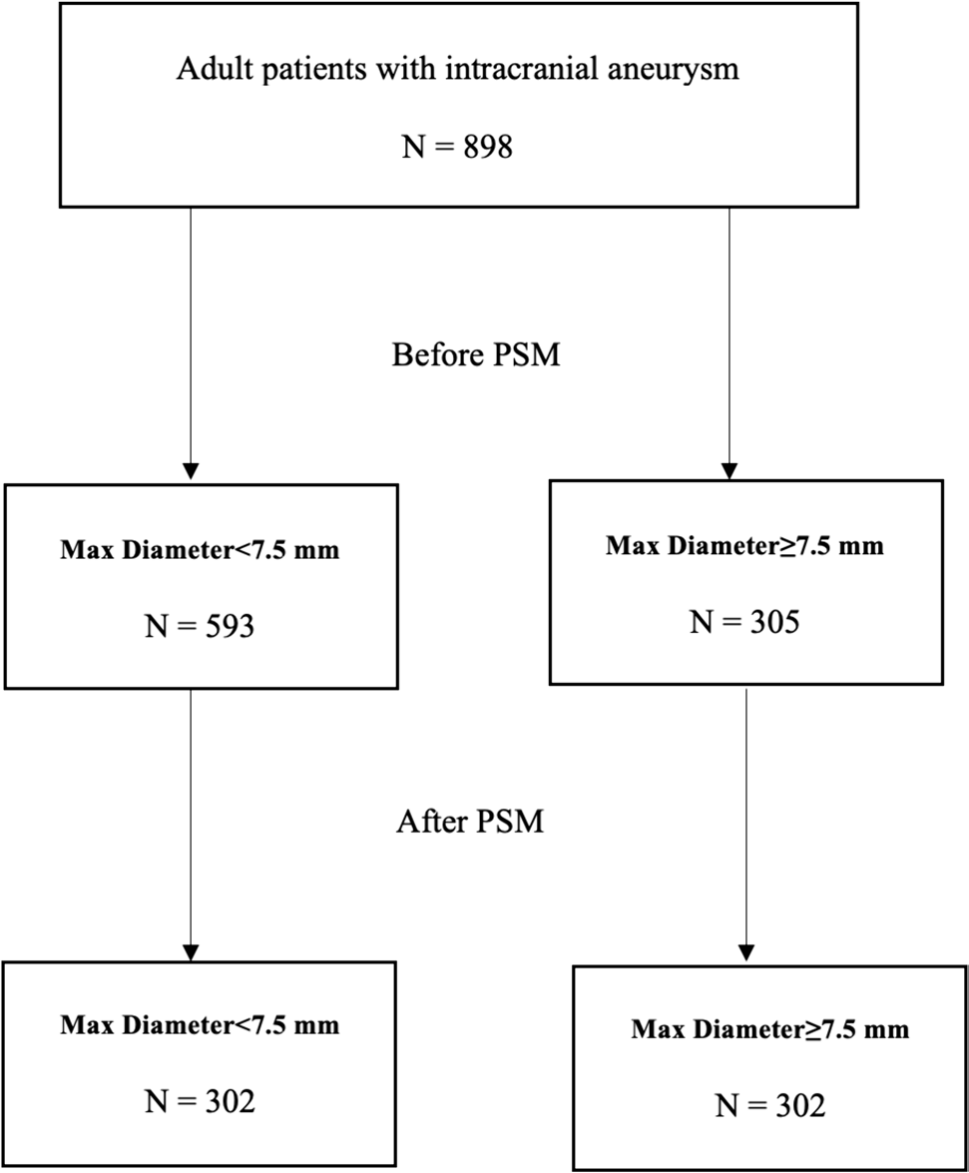
ROC curve analyses for aneurysmal size in relation to retreatment rate

A total of 898 patients were included in this study, with 593 having small aneurysms (maximum diameter < 7.5 mm) and 305 having large aneurysms (≥7.5 mm) (Fig. 2). Most aneurysms in both groups were located in the anterior circulation (82.4% in small vs 75.4% in large) (*p* = 0.009). There was no significant difference observed in clinical presentations (cranial nerve palsy, headache/dizziness, recurrence, seizures, weakness/numbness), smoking status, pretreatment mRS, presence of multiple aneurysms, bifurcation location, or prior treatment (Table 1).

**Table 1:** Comparison of baseline characteristics between small and large aneurysms before and propensity score matching

Variable	Before PSM			After PSM		
	Max Diameter < 7.5 mm, *N* = 593 (66%)^*1*^	Max Diameter ≥ 7.5 mm, *N* = 305 (34%)^*1*^	P^*2*^	Max Diameter < 7.5 mm, *N* = 302 (50%)^*1*^	Max Diameter ≥ 7.5 mm, *N* = 302 (50%)^*1*^	P^*2*^
Gender			0.45			0.32
Female	434 (73)	216 (71)		225 (75)	214 (71)	
Male	159 (27)	89 (29)		77 (25)	88 (29)	
Age (years)	61 (52, 68)	63 (56, 71)	**0.004**	62 (56, 71)	63 (56, 71)	0.97
Smoking Status			0.68			0.74
Current	179 (35)	92 (32)		78 (29)	91 (32)	
Former	146 (28)	83 (29)		82 (30)	82 (29)	
Never	189 (37)	113 (39)		111 (41)	113 (40)	
Presentation Type			0.26			0.97
CN Palsy	7 (1.2)	8 (2.6)		7 (2.3)	8 (2.6)	
Headache/Dizziness	80 (13)	43 (14)		43 (14)	43 (14)	
Incidental/Asymptomatic	488 (82)	239 (78)		240 (79)	236 (78)	
Recurrence	5 (0.8)	7 (2.3)		4 (1.3)	7 (2.3)	
Seizures	5 (0.8)	3 (1.0)		3 (1.0)	3 (1.0)	
Weakness/Numbness	8 (1.3)	5 (1.6)		5 (1.7)	5 (1.7)	
Pre-treatment Modified Rankin Scale			0.92			0.55
0	454 (82)	241 (81)		236 (82)	239 (81)	
1	66 (12)	35 (12)		36 (13)	35 (12)	
2	21 (3.8)	12 (4.0)		12 (4.2)	12 (4.1)	
3	10 (1.8)	8 (2.7)		3 (1.0)	8 (2.7)	
4	1 (0.2)	1 (0.3)		1 (0.3)	0 (0)	
5	1 (0.2)	0 (0)				
Aneurysm Location			**0.009**			0.92
Anterior cerebral artery	206 (35)	80 (26)		88 (29)	80 (26)	
Vertebrobasilar artery	103 (17)	74 (24)		64 (21)	72 (24)	
Internal carotid artery	103 (17)	43 (14)		44 (15)	43 (14)	
Middle cerebral artery	180 (30)	107 (35)		105 (35)	106 (35)	
Posterior cerebral artery	1 (0.2)	1 (0.3)		1 (0.3)	1 (0.3)	
Bifurcation Aneurysm	455 (81)	237 (78)	0.41	232 (79)	234 (78)	0.72
Multiple Aneurysms	191 (34)	112 (38)	0.31	107 (37)	111 (38)	0.88
Prior Treatment	37 (6.4)	23 (7.8)	0.44	18 (6.2)	23 (7.9)	0.42
Aneurysm Neck Size (mm)	3.50 (3.00, 4.20)	5.00 (4.00, 6.00)	** < 0.001**	3.60 (3.00, 4.30)	5.00 (4.00, 6.00)	** < 0.001**
Maximum Aneurysm Diameter (mm)	6.00 (5.00, 6.00)	9.00 (8.00, 10.00)	** < 0.001**	6.00 (5.00, 7.00)	9.00 (8.00, 10.00)	** < 0.001**
Aneurysm Height (mm)	5.00 (4.00, 6.00)	8.00 (7.00, 9.60)	** < 0.001**	5.00 (4.10, 6.00)	8.00 (7.00, 9.63)	** < 0.001**
Aneurysm Width (mm)	4.80 (4.00, 5.70)	7.60 (6.40, 8.80)	** < 0.001**	5.00 (4.00, 5.88)	7.60 (6.40, 8.88)	** < 0.001**
Secondary Aneurysm	114 (22)	84 (29)	**0.018**	83 (30)	82 (29)	0.74
Branch Arising from Aneurysm	53 (9.5)	50 (17)	**0.002**	44 (15)	47 (16)	0.83

^*1*^ *n* (%); Median (IQR)

^*2*^ Pearson’s Chi-squared test; Wilcoxon rank sum test; Fisher’s exact test

The median maximum diameter, neck size, dome height and width were 6 mm, 3.5 mm, 5 mm, and 4.8 mm, respectively, in the small aneurysms group, and 9 mm, 5 mm, 8 mm, and 7.6 mm in the large aneurysms group, which was statistically significant (*p* < 0.001). Aneurysm width did not exceed 10 mm in both groups (Table 1). The large aneurysms group had a higher rate of secondary aneurysms compared to small aneurysms (29% vs 22%, *p* = 0.018).

### Treatment and outcomes

Femoral access was the most common approach used in most aneurysms (Table 2). The median length of angiographic follow-up was 14 months in the large aneurysm group and 15 months in the small aneurysm group (*p* = 0.97). At the last follow-up, adequate aneurysm occlusion was significantly higher in the small aneurysm group (90%, 431/477) compared to large aneurysms (81%, 214/264) (*p* < 0.001). The rate of retreatment was significantly higher in the large aneurysms group (12%) compared to small aneurysms (3.7%) (*p* < 0.001) (Table 2).

**Table 2:** treatment outcomes of small and large aneurysms before and after propensity score matching

Variable	Before PSM			After PSM		
	Max Diameter < 7.5 mm, *N* = 593 (66%)^*1*^	Max Diameter ≥ 7.5 mm, *N* = 305 (34%)^*1*^	P^*2*^	Max Diameter < 7.5 mm, *N* = 302 (50%)^*1*^	Max Diameter ≥ 7.5 mm, *N* = 302 (50%)^*1*^	P^*2*^
WEB Device Type			0.16			0.7
DL	16 (2.8)	14 (4.8)		10 (3.5)	13 (4.5)	
SL	492 (86)	239 (82)		234 (81)	237 (82)	
SLS	64 (11)	40 (14)		45 (16)	40 (14)	
Access Route			0.087			0.63
Femoral	512 (86)	254 (83)		254 (84)	253 (84)	
Radial	81 (14)	49 (16)		48 (16)	47 (16)	
Ulnar	0 (0)	2 (0.7)		0 (0)	2 (0.7)	
Thromboembolic Complications	21 (3.5)	18 (5.9)	0.1	12 (4.0)	18 (6.0)	0.26
Timing of Thromboembolic Complications			0.84			0.88
Intraop	10 (48)	8 (44)		5 (42)	8 (44)	
Postop	11 (52)	10 (56)		7 (58)	10 (56)	
Duration of Thromboembolic Complications			0.71			> 0.99
Permanent	5 (38)	5 (31)		3 (33)	5 (31)	
Transient	8 (62)	11 (69)		6 (67)	11 (69)	
Hemorrhagic Complications	6 (1.1)	6 (2.0)	0.36	3 (1.0)	6 (2.1)	0.5
Timing of Hemorrhagic Complications			0.57			0.17
Intraop	4 (67)	2 (33)		3 (100)	2 (33)	
Postop	2 (33)	4 (67)		0 (0)	4 (67)	
Duration of Hemorrhagic Complications			> 0.99			> 0.99
Permanent	2 (33)	2 (40)		1 (33)	2 (40)	
Transient	4 (67)	3 (60)		2 (67)	3 (60)	
Other Complications	30 (5.9)	19 (6.9)	0.57	16 (6.0)	19 (6.9)	0.67
Type of Other Complications			0.9			0.82
Access site complication	1 (4.5)	1 (9.1)		0 (0)	1 (9.1)	
Air embolus	1 (4.5)	0 (0)				
Contrast reaction	2 (9.1)	0 (0)				
Deployment issue	7 (32)	5 (45)		5 (45)	5 (45)	
Groin Hematoma	1 (4.5)	1 (9.1)		0 (0)	1 (9.1)	
Hematoma/Pseudoaneurysm	10 (45)	4 (36)		6 (55)	4 (36)	
Duration of Other Complications						
Transient	11 (100)	8 (100)		6 (100)	8 (100)	
Antiplatelet Therapy	520 (88)	246 (81)	**0.004**	266 (88)	244 (81)	**0.017**
Last Clinical Follow-Up	12 (5, 23)	13 (6, 27)	0.083	11 (5, 20)	13 (6, 27)	**0.036**
Last Modified Rankin Scale			0.2			0.18
0	438 (80)	220 (76)		219 (79)	218 (76)	
1	74 (13)	39 (13)		39 (14)	39 (14)	
2	18 (3.3)	10 (3.5)		11 (4.0)	10 (3.5)	
3	9 (1.6)	7 (2.4)		3 (1.1)	7 (2.4)	
4	4 (0.7)	2 (0.7)		4 (1.4)	2 (0.7)	
5	1 (0.2)	1 (0.3)		0 (0)	1 (0.3)	
6	5 (0.9)	10 (3.5)		2 (0.7)	10 (3.5)	
Last mRS 0–1	512 (93)	259 (90)	0.065	258 (93)	257 (90)	0.17
Last mRS 0–2	530 (97)	269 (93)	**0.024**	269 (97)	267 (93)	**0.044**
Last mRS 6 (Mortality)	5 (0.9)	10 (3.5)	**0.008**	2 (0.7)	10 (3.5)	**0.023**
Last Imaging Follow-Up	15 (6, 24)	14 (6, 25)	0.97	14 (6, 22)	14 (6, 24)	0.57
Immediate Flow Stagnation	505 (90)	276 (92)	0.34	265 (92)	273 (92)	0.92
Immediate Raymond-Roy Classification			**0.004**			0.17
1	176 (31)	60 (21)		77 (27)	60 (21)	
2	115 (20)	69 (24)		56 (20)	68 (24)	
3	272 (48)	162 (56)		149 (53)	160 (56)	
Last Follow-Up Raymond-Roy Classification			** < 0.001**			** < 0.001**
1	317 (66)	125 (47)		168 (70)	125 (48)	
2	114 (24)	89 (34)		48 (20)	87 (33)	
3	46 (9.6)	50 (19)		25 (10)	50 (19)	
Adequate Occlusion (RR1 + RR2)	431 (90)	214 (81)	** < 0.001**	216 (90)	212 (81)	**0.006**
Inadequate Occlusion (RR3)	46 (9.6)	50 (19)	** < 0.001**	25 (10)	50 (19)	**0.006**
Compaction			0.29			**0.034**
Same	225 (59)	122 (54)		128 (65)	122 (54)	
Minor	123 (32)	75 (33)		56 (28)	73 (33)	
Major	35 (9.1)	29 (13)		13 (6.6)	29 (13)	
Major Compaction	35 (9.1)	29 (13)	0.15	13 (6.6)	29 (13)	**0.03**
Retreatment Required	22 (3.7)	37 (12)	** < 0.001**	11 (3.6)	37 (12)	** < 0.001**
Type of Retreatment			**0.047**			**0.013**
Clipping	2 (9.1)	2 (5.4)		0 (0)	2 (5.4)	
Coiling	4 (18)	6 (16)		1 (9.1)	6 (16)	
Contour	1 (4.5)	0 (0)				
Endovascular techniques	5 (23)	1 (2.7)		4 (36)	1 (2.7)	
FD	4 (18)	5 (14)		3 (27)	5 (14)	
SAC	5 (23)	21 (57)		2 (18)	21 (57)	
WEB	1 (4.5)	2 (5.4)		1 (9.1)	2 (5.4)	

^*1*^ *n* (%); Median (IQR)

^*2*^ Pearson’s Chi-squared test; Wilcoxon rank sum test; Fisher’s exact test

The majority of patients had a mRS of 0 at last follow-up, and a comparison in mRS scores is reported in Table 2. There was no significant difference observed between the two groups in terms of thromboembolic complications (*p* = 0.1), and hemorrhagic complications (*p* = 0.36) (Table 2).

### Propensity score matching

PSM was used to match both groups by age, gender, smoking status, presenting signs/symptoms, pretreatment mRS, bifurcation location, presence of multiple aneurysms, previous treatment, daughter sacs, and the presence of aneurysmal branch. This resulted in 302 matched pairs (Tables 1 and 2).

After PSM, the large aneurysm group had a significantly lower adequate occlusion rate at last follow-up compared to the small aneurysm group (81%, 212/262, vs. 90%, 216/241, *p* = 0.006). Retreatment was needed more frequently in the large aneurysm group (12%) compared to the small aneurysm group (3.6%) (*p* < 0.001).

In terms of the mRS score at last follow-up, patients in the large aneurysm group achieved lower rates of good functional outcomes (mRS 0–2) compared to the patients in the small aneurysm group (93% vs. 97%, *p* = 0.044). The same was observed regarding mortality rates where patients in the large aneurysm group had a higher mortality rate compared to those in the small aneurysm group (3.5% vs. 0.7%, *p* = 0.023) There were no significant differences observed in thromboembolic complications, hemorrhagic complications, or any of the other complications rates between the two groups.

## Discussion

The aim of this multicenter cohort study was to compare the angiographic and clinical outcomes between small (maximum diameter < 7.5 mm) and large (≥7.5 mm) aneurysms using the WEB device. After matching patient and aneurysms characteristic between both groups, small aneurysms were associated with significantly higher rate of adequate occlusion at last follow-up (90%. vs. 81%), and significantly lower rate of retreatment. (3.6% vs 12%).

Large aneurysms pose a greater threat compared to smaller aneurysms due to increased risk of rupture and more complex treatment procedures [12]. Due to the higher risk of rupture in large aneurysms, early surgical or endovascular treatment may be warranted. Endovascular treatment using coil embolization is technically challenging for large aneurysms due to longer procedure times, low packing density, and high recurrence rates [13–17]. For wide-necked aneurysms, assistive devices like stents or balloons are often needed to prevent coil migration. Chalouhi et al. [1] reported immediate complete aneurysm occlusion of large aneurysms (≥ 10 mm) in 87.6% of aneurysms. Complications occurred in 10.5% of patients, with 1 death (0.3%). Recanalization and retreatment rates were 39% and 33%, respectively. Larger aneurysm size was a predictor of poor outcomes. The effectiveness of using coil embolization to treat large aneurysms is limited due to several factors. Firstly, coils are unable to produce permanent thrombosis, which is necessary for successful treatment. Secondly, there may already be thrombosis present inside the aneurysm at the time of treatment, which can make it challenging to pack the coils effectively. Lastly, coils may not be able to fully reconstruct the endothelial lining of the neck, leading to poor treatment outcomes. Even after a period of 2–6 months following coil treatment, there is often no observed improvement in the thrombus organization or endothelization of the neck [18, 19].

The use of flow diverters has emerged as a highly promising alternative for treatment of sidewall complex aneurysms, particularly in cases where intrasaccular coil embolization may not be suitable [20]. Unlike coil embolization, flow diverters are not limited by the same drawbacks and have been FDA-approved for the treatment of large aneurysms located in the petrous to superior hypophyseal segment of the internal carotid artery [20]. Over time, the scope of their application has expanded to include a wide range of aneurysms, including those that have been previously treated, are acutely ruptured, small-sized, located in the posterior circulation, or classified as non-saccular lesions such as fusiform, dissecting, and pseudoaneurysms [20]. Kim et al. [21] conducted a multicenter study on the treatment of 47 aneurysms with a size of 15 mm or smaller using the Pipeline Embolization Device (PED). According to their report, 77.4% of aneurysms achieved complete occlusion after a median follow-up of 3 months. However, treatment-related morbidity was observed in 4.4% of cases, with ischemic stroke being the main cause. The use of flow diversion stents is limited in bifurcation aneurysms due to concerns of branch occlusion and thromboembolic complications.

The WEB device is a new type of intrasaccular flow disruptor that has been developed specifically to treat wide-necked bifurcation aneurysms in certain cases without the need for dual antiplatelet therapy [22, 23]. This has been demonstrated in studies such as the WEB-IT trial, where only 11% of patients were using dual antiplatelet therapy at the 6-month follow-up visit [22]. However, large aneurysms with width greater than 10 mm cannot typically be treated using WEB, given the current maximum size of 11 × 9.6 mm and adequate lateral wall-apposition is critical to ensure flow disruption at the aneurysm neck [24]. However, this has not stopped the off-label use of WEB for treatment of large aneurysms with maximum diameter of ≥10 mm if the width remains within the available device size. A study was conducted by Khalid et al. [10] to evaluate the efficacy of WEB in large, complex intracranial aneurysms. A total of 16 patients were included. The mean aneurysm size was 11.3 ± 1.7 and the median follow-up was 36 months. Aneurysms were predominantly located at the basilar artery bifurcation and anterior communicating artery. Three out of sixteen aneurysms were ruptured. Despite achieving complete occlusion immediately in 75% of intracranial aneurysms, 7 out of 15 cases (46.7%) required retreatment, mainly due to increasing neck remnants and recurrences after 1-year follow-up [10].

In the present study, the use of WEB in the small and large aneurysm groups showed a significant difference in the rates of complete occlusion at the last follow-up, with the small aneurysm group having higher rates of occlusion. The retreatment rates were also significantly different, with the small aneurysm group having lower retreatment rates.

### Limitations

Our study has a few limitations that must be considered. Firstly, since it is a retrospective study, there is a possibility of incomplete data sets, selection bias, and unidentified confounders. To counteract this, we utilized PSM to balance the two groups and minimize the risk of selection bias. However, it is important to note that PSM only controls for measured confounders, not unmeasured ones, and thus does not replace randomization. Secondly, as our study included multiple institutions, there may be variability in patient management, aneurysm measurement, and device compaction rate. Nonetheless, the use of a standardized data sheet across all centers and a large sample size may increase the generalizability of the findings. However, it is worth mentioning that we lacked a core laboratory to objectively evaluate the radiologic outcomes of the degree of aneurysm occlusion.

## Conclusion

The current study provides valuable insights into the differences between large and small aneurysms in terms of patient and aneurysmal characteristics, treatment approaches, and outcomes. The results showed a significantly lower occlusion rate and higher retreatment rate in large aneurysms compared to small aneurysms. These findings may help guide treatment decisions and patient counseling.

## Data Availability

No datasets were generated or analysed during the current study.
